# Pre-existing cardiovascular disease increases risk of atrial arrhythmia and mortality in cancer patients treated with Ibrutinib

**DOI:** 10.1186/s40959-021-00125-8

**Published:** 2021-11-19

**Authors:** Juan Carlo Avalon, Jacob Fuqua, Tyler Miller, Seth Deskins, Chelby Wakefield, Austin King, Sonya Inderbitzin-Brooks, Christopher Bianco, Lauren Veltri, Wei Fang, Michael Craig, Abraham Kanate, Kelly Ross, Midhun Malla, Brijesh Patel

**Affiliations:** 1 West Virginia University School of Medicine, Morgantown, USA; 2 West Virginia University Heart and Vascular Institute, Morgantown, WV 26506 USA; 3 West Virginia University Mary Babb Randolph Cancer Institute, Morgantown, USA; 4 West Virginia Clinical and Translational Science Institute, Morgantown, USA

**Keywords:** Ibrutinib, Atrial fibrillation, Cardio-oncology, Cardiovascular disease, Arrhythmia, Hematologic malignancy

## Abstract

**Background:**

Ibrutinib is a Bruton’s tyrosine kinase inhibitor used in the treatment of hematological malignancies. The most common cardiotoxicity associated with ibrutinib is atrial arrhythmia (atrial fibrillation and flutter). It is known that patients with cardiovascular disease (CVD) are at an increased risk for developing atrial arrhythmia. However, the rate of atrial arrhythmia in patients with pre-existing CVD treated with ibrutinib is unknown.

**Objective:**

This study examined whether patients with pre-existing CVD are at a higher risk for developing atrial arrhythmias compared to those without prior CVD.

**Methods:**

A single-institution retrospective chart review of patients with no prior history of atrial arrhythmia treated with ibrutinib from 2012 to 2020 was performed. Patients were grouped into two cohorts: those with CVD (known history of coronary artery disease, heart failure, pulmonary hypertension, at least moderate valvular heart disease, or device implantation) and those without CVD. The primary outcome was incidence of atrial arrhythmia, and the secondary outcomes were all-cause mortality, risk of bleeding, and discontinuation of ibrutinib. The predictors of atrial arrhythmia (namely atrial fibrillation) were assessed using logistic regression. A Cox-Proportional Hazard model was created for mortality.

**Results:**

Patients were followed for a median of 1.1 years. Among 217 patients treated with ibrutinib, the rate of new-onset atrial arrhythmia was nearly threefold higher in the cohort with CVD compared to the cohort without CVD (17% vs 7%, *p* = 0.02). Patients with CVD also demonstrated increased adjusted all-cause mortality (OR 1.9, 95% CI 1.06-3.41, *p* = 0.01) and decreased survival probability (43% vs 54%, *p* = 0.04) compared to those without CVD over the follow-up period. There were no differences in risk of bleeding or discontinuation between the two cohorts.

**Conclusions:**

Pre-existing cardiovascular disease was associated with significantly higher rates of atrial arrhythmia and mortality in patients with hematological malignancies managed with ibrutinib.

## Introduction

Ibrutinib is a Bruton’s tyrosine kinase (BTK) inhibitor that is commonly used for the treatment of various hematological malignancies since first approved in November 2013 [1]. Till date, this drug has been approved and indicated for treatment of chronic lymphocytic leukemia (CLL)/small lymphocytic lymphoma (SLL), mantle cell lymphoma, marginal zone lymphoma, Waldenstrom macroglobulinemia, and chronic graft versus host disease (GvHD) [1–5].

As with many chemotherapeutic agents, ibrutinib has a well-documented side effect profile including bleeding, infections, cytopenias, hypertension, tumor lysis syndrome, and cardiac arrhythmias [6–12]). Common cardiac arrhythmias that have been associated with ibrutinib include ventricular tachyarrhythmias and, most commonly, atrial fibrillation. Atrial fibrillation has been documented in 4% of patients in clinical trials and 5.77 per 100 person-years while another study has demonstrated an 11% incidence of atrial arrhythmia over a 5-year follow-up [13, 14]. Ibrutinib has an off-target inhibitory effect on Tec protein tyrosine kinase (TEC). Both BTK and TEC are expressed in cardiac tissue with an increased concentration in atrial tissue [15–17].

Development of atrial fibrillation is also associated with multiple modifiable risk factors including obesity, hypertension, diabetes, obstructive sleep apnea, alcohol consumption, smoking, and sedentary lifestyles [18–21]. Additionally, coronary artery disease (CAD) has also been associated with atrial fibrillation, with the prevalence of CAD in patients with atrial fibrillation ranging from 17 to 46.5% [19]. Although some of these risk factors have a more pronounced contribution to the incidence of atrial fibrillation, ibrutinib was shown to increase the incidence of atrial fibrillation independently of these risk factors [22]. Since both cardiovascular disease and the use of ibrutinib are associated with an escalated risk for atrial fibrillation, our study aimed to investigate the incidence of new-onset atrial fibrillation after ibrutinib initiation in patients with underlying cardiovascular disease (CVD).

## Methods

Approval and appropriate oversight from the Institutional Board Review was obtained prior to data collection. We retrospectively collected data on consecutive patients who received ibrutinib from November 2012 to September 2020 at a single institution. This study period was chosen because it was the first initiation date of ibrutinib for a patient to the last known follow-up. This study’s primary outcome was to assess if prior CVD increased the risk of atrial fibrillation incidence after receiving ibrutinib. The secondary outcomes were all-cause mortality, bleeding, and discontinuation of Ibrutinib. We divided the patients into two cohorts 1) patients with prior cardiovascular disease (CVD) and 2) patients without CVD. Patients with known CAD (with or without revascularization), known chronic heart failure with reduced ejection fraction or chronic heart failure with preserved ejection fraction, pulmonary hypertension, pacemakers, implantable cardioverter defibrillators, ventricular arrhythmia, or at least moderate valvular heart disease were included in the prior CVD group. Since one of the outcomes of interest was a new diagnosis of atrial fibrillation, we excluded patients with an existing diagnosis of atrial fibrillation before ibrutinib initiation. We excluded patients with missing data for medical history or the ibrutinib initiation date. We also excluded patients who received Ibrutinib for GVHD because their clinical characteristics would be heterogenous than patients with other malignancies. The clinical variables are either self-reported by the patients, documented diagnoses from clinicians, or confirmed through review of relevant cardiac testing including, but not limited to, electrocardiogram (ECG) or echocardiography.

Categorical variables were analyzed with the chi-square test and expressed as percentages. The continuous variables were analyzed with the Mann-Whitney U test after evaluating the normalcy of data (using the Shapiro-Wilk test) and expressed as medians and interquartile range. A binary logistic regression was created to predict CVD as an independent predictor for new-onset atrial fibrillation after adjusting for age, chronic kidney disease, hypertension, and initiation dose of the medications, since these variables are closely linked to the atrial fibrillation and can be disproportionately higher in the CVD group. The finding was reported as an odds ratio with a 95% confidence interval. We also created a Cox proportional hazards model to factor in age, cancer diagnoses, post-chemotherapy, and atrial fibrillation for CVD to predict mortality expressed as hazard ratio with 95% confidence intervals. We selected these variables for this model since they can strongly influence the mortality, especially in the CVD group. The follow-up time for this analysis was calculated from initiation to the last known follow-up date in the chart. The survival probability after checking for assumptions was estimated using the Kaplan-Meier curve and the log-rank test was used to compare the prior CVD and without CVD groups. The statistical analysis was conducted on SPSS (IBM Corp., Amrock, New York) and SAS 9.4 (SAS Institute, Cary, North Carolina).

## Results

We identified 300 patients taking ibrutinib from November 2012 to September 2020. Of these, 51 patients had missing data for medical history, 7 patients had GvHD, 18 patients had missing initiation dates, and 7 patients had atrial fibrillation before chemotherapy initiation. After excluding these patients, the final cohort included 217 patients for further analysis. The follow-up time was available for 189 patients (87%), and the median follow-up time was 1.1 years, with an IQR of 0.4 to 2.6 years. The entire cohort’s median age was 74 years (IQR: 64-81 years). Males comprised 64% of the entire cohort and majority of the patients were White (93%). Chronic lymphocytic leukemia (CLL) and small lymphocytic lymphoma (SLL) were the most common forms of cancer (72%), followed by mantle cell lymphoma (16%) and Waldenstrom macroglobulinemia (5%). Marginal zone lymphoma, diffuse large B-cell lymphoma, and other types of hematologic malignancies constituted the remaining 6%.

### Baseline characteristics

Prior CVD cohort had 69 patients (32%) and without CVD cohort had 148 patients (68%).

Patients with known CAD (with [12%] or without [6.5%] revascularization), known chronic heart failure with reduced ejection fraction (4.6%) or chronic heart failure with preserved ejection fraction (10.1%), pulmonary hypertension (1%), pacemakers (1.4%), implantable cardioverter defibrillators (0.5%), ventricular arrhythmia (1.8%), or at least moderate valvular heart disease (8.3%) were included in the prior CVD group. The median ages for the prior CVD and without CVD cohorts were 74 and 70 years, respectively (*p* = 0.02). There were no statistical differences in gender or race between the two cohorts. The patients with prior CVD had a higher burden of comorbidities such as hypertension, diabetes, dyslipidemia, and chronic obstructive lung disease (Table 1). There was no statistical difference for cerebrovascular events, liver disease, chronic kidney disease, tobacco use, or illicit drug use between the two cohorts. Higher proportions of patients in the prior CVD cohort were on statins, neurohormonal blocking drugs (e.g., beta blockers, angiotensin-converting enzyme inhibitors), and aspirin. However, there was no statistical difference for CYP3A inhibitors or inducers, anticoagulants, or diuretics between the two groups (Table 1).

**Table 1:** Baseline characteristics of ibrutinib patients

Variables	Without CVD^a^	CVD	Total	***p***-value
	*n* = 148 (68%)	*n* = 69 (32%)	*N* = 217	
**Demographics**				
Age	70 (63-76)	74 (64-80)	74 (64-81)	0.02
BMI	27 (24-31)	27 (24-31)	27 (23-32)	0.90
Male	63	65	64	0.73
White	93	94	93	0.26
Black	3	0	2	
Unknown/Other	4	6	5	
**Comorbidities**				
Hypertension	58	83	66	< 0.001
Diabetes	18	32	23	0.03
Dyslipidemia	41	67	49	< 0.001
Chronic obstructive lung disease	14	26	18	0.023
Cerebrovascular accidents	3	6	4	0.26
Chronic kidney disease	10	16	12	0.22
Liver disease	6	6	6	0.96
Alcohol use	26	25	26	0.79
Tobacco use	53	48	51	0.50
Illicit drug use	3	1	3	0.42
**Medications**				
Statins	28	54	36	< 0.0001
Beta blockers	21	44	28	0.001
Neurohormal blocking agents	29	45	34	0.02
Spironolactone	1	1	1	0.95
Aspirin	17	52	28	< 0.001
Warfarin	1	3	1	0.19
Direct oral anticoagulants	7	1	5	0.10
Calcium channel blockers	14	16	14	0.63
Diuretics	20	30	24	0.10
Sodium-glucose cotransporter 2	1	3	2	0.43
Prior chemotherapy use	43	42	43	0.83
CYP3A inhibitors	10	12	10	0.63
CYP3A inducers	0	7	2	0.001
**Initial dose**				
560 mg daily	17	23	19	0.10
420 mg daily	77	64	73	
280 mg daily	6	13	8	
**Cancer diagnoses**				
Mantel cell lymphoma	14	19	16	0.65
CLL^b^/^c^SLL	74	70	72	
Marginal zone lymphoma	1	0	1	
Waldenstrom macroglobunemia	5	3	5	
Diffuse large B-cell lymphoma	3	3	3	
Others	2	3	2	

^a^Cardiovascular disease; ^b^Chronic lymphocytic leukemia; ^c^Small lymphocytic lymphoma

#### Outcomes

**Fig. 1:**
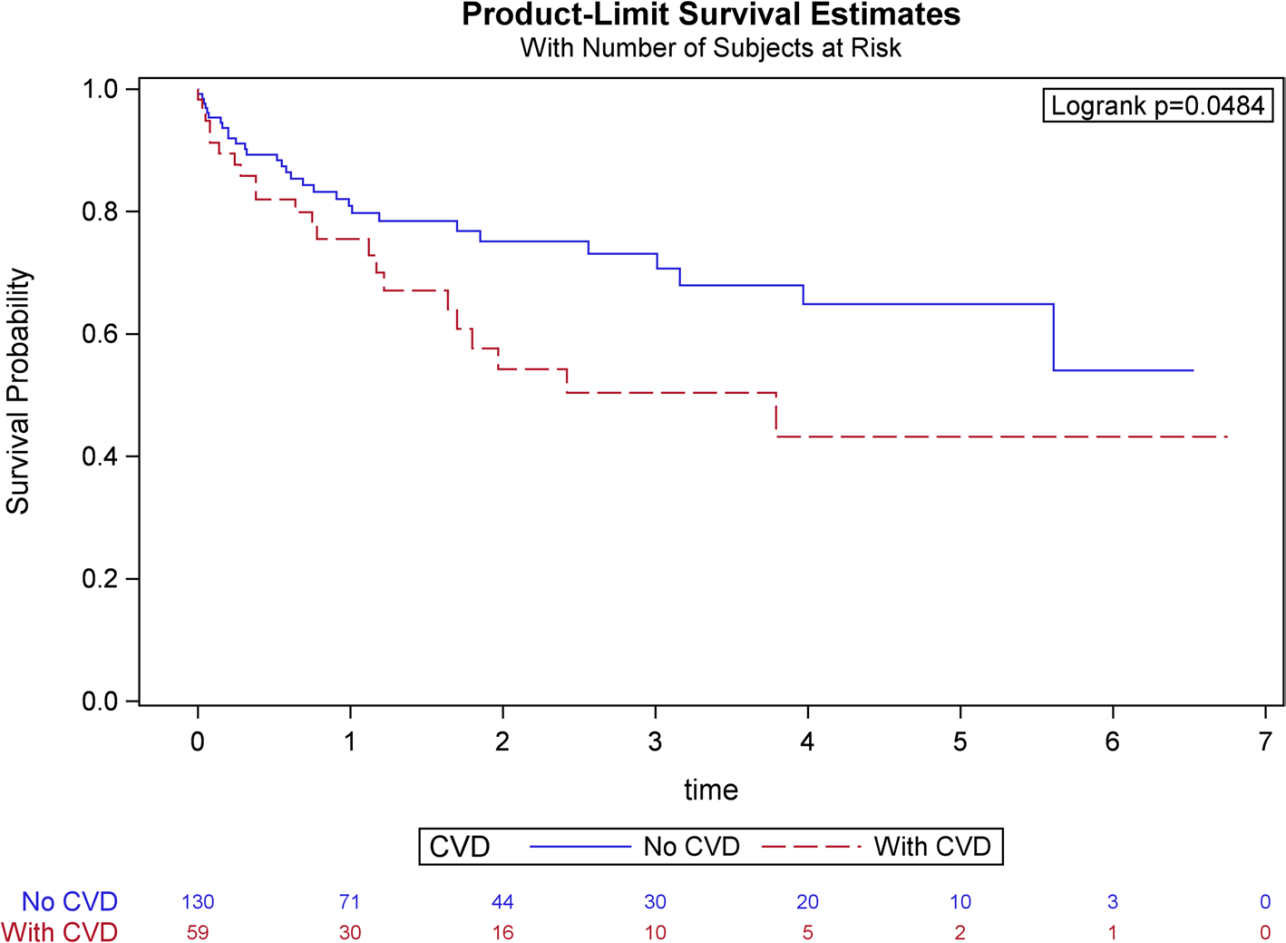
Cox-proportional Hazard model showing increased hazard ratio associated with the presence of cardiovascular disease. Caption: CVD = cardiovascular disease

The primary outcome of interest was new-onset atrial fibrillation. Twelve patients (17.4%) with prior CVD compared to 10 patients (6.8%) without CVD developed atrial fibrillation (*p* < 0.02), indicating CVD status was associated with new-onset atrial fibrillation. After adjusting for age, hypertension, chronic kidney disease, and Ibrutinib initiation dose, those with prior CVD have 2.91 times the odds (95% confidence interval: 1.19-7.25; *p* = 0.02) of developing atrial fibrillation than those without prior CVD. Secondary outcomes including bleeding (19% with prior CVD vs. 16% without CVD; *p* = 0.65) and discontinuation of ibrutinib (44% with prior CVD vs. 31% without CVD; *p* = 0.08) were not statistically significant between the two cohorts, indicating CVD status was not significantly associated with either bleeding events or discontinuation of ibrutinib. However, the mortality rate was significantly higher in patients with prior CVD (39%) vs. without CVD (23%) (*p* = 0.01) over the follow-up period (Table 2). After adjusting for the following confounders age, new-onset atrial fibrillation, and cancer diagnoses (CLL/SLL and other cancers combined were treated as two groups), a hazard ratio for mortality with CVD as a predictor was 1.90 (95% confidence interval: 1.06-3.41). The estimated survival probability was 43% with prior CVD and 54% without CVD over the follow-up period (log-rank test *p* = 0.0484) (Fig. 1).

**Table 2:** Primary and secondary outcomes

Outcomes	Without CVD	CVD	Total	*p*-value	Odds ratio (95% Confidence interval)
Afib^a^	7	17	10	0.02	Adjusted: 2.91 (1.19-7.25)
Discontinuation	31	44	35	0.08	Unadjusted: 1.71 (0.95-3.08)
Bleeding	16	19	17	0.65	Unadjusted: 1.20 (0.55-2.61)
					**Hazards ratio (95% Confidence interval)**
Mortality^b^	23	39	28	0.01	Adjusted: 1.90 (1.06-3.41)

^a^Adjusted for age, hypertension, chronic kidney disease, and ibrutinib initiation dose

^b^Adjusted for age, new-onset atrial fibrillation, and cancer diagnoses

## Discussion

Given the widespread use of ibrutinib, patients and clinicians must be aware of the cardiotoxic potential. Atrial fibrillation is the most encountered cardiac arrhythmia and contributes to a myriad of complications. Our single-institution study examined the incidence of new-onset atrial arrhythmia in patients with and without pre-existing CVD treated with ibrutinib for various hematologic malignancies, most commonly CLL. Hypertension, diabetes, hyperlipidemia, and COPD were the most significant comorbidities in our cohort of patients with prior CVD and remain amongst the most common causes of major modifiable risk factors for CVD across the general population (Table 1). While the association between ibrutinib use and the development of atrial fibrillation has been previously cited in the literature as high as 6-16% [22–24], our data demonstrated that patients with pre-existing CVD prior to initiation of ibrutinib were nearly three-times more likely to develop atrial fibrillation than those without prior CVD. A previous study demonstrated that an elevated Framingham Heart Study-AF score was associated with increased incidence of atrial fibrillation in those receiving ibrutinib therapy [25]. Survival probability estimates also differed significantly for those patients with a history of CVD versus those without CVD, with an absolute difference of 11% in those patients with prior CVD vs. those without CVD within our follow-up. Previous studies have demonstrated the time-to-onset of atrial fibrillation after ibrutinib initiation to occur within the first year of ibrutinib initiation [26].

The pathophysiology of atrial fibrillation is multifactorial, and various etiologies have shown association with the development of atrial fibrillation. In particular, cardiac conditions associated with persistent inflammation and ischemia have been implicated in the development of atrial fibrillation. Valvular heart disease, congestive heart failure, and coronary artery disease also increase the likelihood of atrial fibrillation. Elevated filling pressures within the atria, either due to hemodynamically significant valvular disease or diminished cardiac function, leads to eventual chamber dilation and resultant fibrosis. This cardiac remodeling provides the foundation for the electrical disturbances, primarily through ion channel dysfunction and myocyte uncoupling associated with atrial fibrillation [27]. On a molecular level, profibrotic growth factors, such as transforming growth factor-beta1 (TGF-B1) and platelet-derived growth factor/vascular endothelial growth factor (PDGF/VEGF), are upregulated as a result of various cardiac injury [28].

Disruptions in molecular signaling also provide insights into the association of ibrutinib and atrial fibrillation. Through the binding to cysteine 481 residue of BTK, ibrutinib inhibits the dysregulated B-cell receptor signaling responsible for proliferation and survival in B-cell malignancies [29, 30]. BTK is expressed in human cardiac tissue and appears to be expressed greater in patients with atrial fibrillation compared to those in normal sinus rhythm [16, 17], which could explain the pro-arrhythmogenic effects of ibrutinib in atrial dysrhythmias. Similar sequela of atrial dysfunction has been seen in those with genetic mutations of tyrosine kinase pathways, such as the KCNA5 mutation which encodes the ultrarapid delayed rectifier potassium channel that then in turn modulates tyrosine kinase signaling [31]. Other in vivo animal studies have suggested that ibrutinib induces atrial fibrillation through structural remodeling and dysregulated calcium handling within atrial myocytes [32].

Our study demonstrates the increased rate of atrial fibrillation and decreased survival in patients with pre-existing CVD initiated on ibrutinib for hematological malignancies. No formal guidelines exist for how to monitor and treat this population, although some strategies have been proposed through the assessment of hemodynamic stability, ECG and echocardiograph findings, and careful assessment of drug-drug interactions in rate/rhythm control pharmacotherapy [33]. While most studies on the management of atrial fibrillation are in patients without malignancy, cancer patients with atrial fibrillation are at an increased risk of both heart failure and thromboembolism [34]. In addition to anticoagulation medications, rate and rhythm control medications also interact with ibrutinib through the CYP450 CYP3A metabolic pathway, making management of atrial fibrillation difficult [35]. Surveillance electrocardiography in order to identify left atrial abnormalities has been identified as a simple clinical tool, especially in the early stages of treatment [36]. Hypertension is a well-known risk factor for the development of atrial fibrillation in addition to other adverse cardiovascular events, independent of ibrutinib therapy [10, 20]. Routine blood pressure monitoring should also be part of regular surveillance metrics in patients on ibrutinib therapy, especially those with pre-existing hypertension [37, 38]. One study from Roeker et al. revealed that patients on ibrutinib therapy experienced increased systolic and diastolic blood pressure readings within the first year of ibrutinib initiation, with up to 21 mmHg median change in systolic blood pressure and 8.5 mmHg in diastolic blood pressure [39]. Clinicians should be aware of this effect in order to mitigate the potential for new onset or worsening hypertension in patients on ibrutinib, with special attention to the acute post-initiation period. More studies are warranted to help create sound clinical guidelines for the management of atrial fibrillation in patients being treated with ibrutinib with careful consideration to stroke risk and other cardiac complications.

### Limitations

Our study was limited due to a relatively small sample size and inherent nature of retrospective studies. Additionally, our search for patients on ibrutinib was limited to our institutional EMR only. From the chart review, it was difficult to locate exact dates or timing of atrial fibrillation, and “time-to-event” was not possible. Our decision to include patients with various cardiac conditions was based on our referral patterns, but future studies should focus on the impact of specific cardiovascular conditions on ibrutinib patients. Many echocardiographic variables, such as atrial size, are not available since the majority of patients did not undergo routine echocardiography before and while on ibrutinib. Therefore, we couldn’t adjust for these variables.

Our study highlights important implications for management of patients on Ibrutinib. While our study did not reveal statistically significant differences in bleeding events between the two cohorts, predisposition to bleeding is a well-known side effect of ibrutinib [40, 41]. This must be taken into consideration when either initiating, continuing, or terminating anticoagulation therapy for stroke and thromboembolic prevention in patients receiving ibrutinib therapy, as certain groups of patients may already be receiving antiplatelet therapy. This also presents a clinical conundrum in the decision of whether to discontinue ibrutinib at the expense of progressive oncologic disease or to continue ibrutinib treatment with the risk of cardiovascular compromise. Our study did not reveal a difference in the rate of ibrutinib discontinuation in our two cohorts; however, atrial fibrillation remains the most common cause of ibrutinib discontinuation in treated patients [42, 43].

## Conclusions

**Fig. 2:**
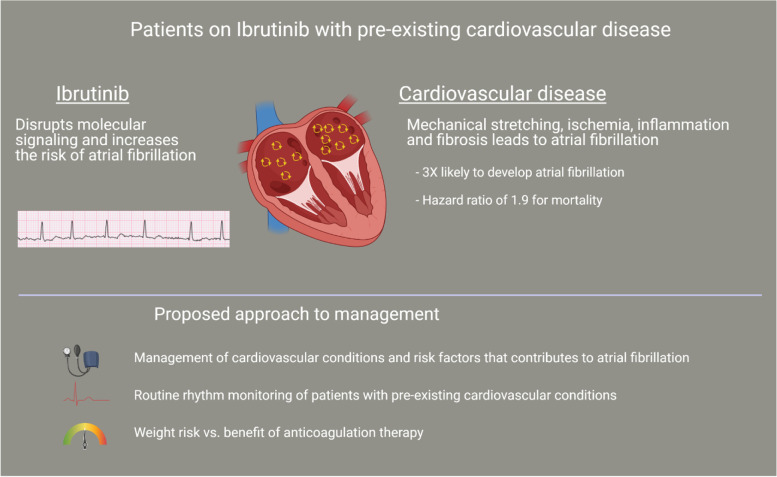
The patient with concomitant cardiovascular disease receiving Ibrutinib are more likely to develop atrial fibrillation. This owes to both underlying cardiovascular condition related changes as well as the disruption in molecular signaling. We propose that the patients should be screened and managed for pre-existing cardiovascular conditions prior to initiating Ibrutinib. Atrial fibrillation can be asymptomatic and associated with increased risk of thromboembolic events. And, therefore, we recommend rhythm screening for patients with cardiovascular conditions

Cardio-oncology has emerged as a quickly growing specialty to help manage patients with malignancy and cardiovascular comorbidities. We highly encourage hematology-oncologists to work together with cardio-oncologists in a multi-disciplinary setting for effective management of patients on ibrutinib therapy. Through early identification of cardiac disease, assessment of cardiovascular structure and function, optimization of cardiovascular risk factors, and the utilization of surveillance testing such as serial electrocardiogram during treatment (Fig. 2), cardio-oncologists play a vital role in the prevention and management of cardiotoxicity in cancer patients.

## Data Availability

The datasets used and/or analyzed during the current study are available from the corresponding author on reasonable request. This is subject to the institutional data sharing policy.

## Abbreviations

CVD: Cardiovascular disease
AF: Atrial fibrillation
GvHD: Graft versus host disease
CLL: Chronic lymphocytic leukemia
BTK: Bruton’s tyrosine kinase
ECG: electrocardiogram
CAD: Coronary artery disease
CI: Confidence interval
